# Multilayer Structure Damage Detection Using Optical Fiber Acoustic Sensing and Machine Learning

**DOI:** 10.3390/s24175777

**Published:** 2024-09-05

**Authors:** Beatriz Brusamarello, Uilian José Dreyer, Gilson Antonio Brunetto, Luis Fernando Pedrozo Melegari, Cicero Martelli, Jean Carlos Cardozo da Silva

**Affiliations:** 1 Graduate Program in Electrical and Computer Engineering (CPGEI-UTFPR), Curitiba 80230-901, Brazil; beatrizbrusamarello@alunos.utfpr.edu.br (B.B.); uiliandreyer@utfpr.edu.br (U.J.D.); cmartelli@utfpr.edu.br (C.M.); 2 CPFL Energia, Campinas 13076-970, Brazil; gilson.brunetto@cpfl.com.br (G.A.B.); lmelegari@cpfl.com.br (L.F.P.M.)

**Keywords:** damage detection, distributed acoustic sensing, machine learning, optical fiber sensors, structural health monitoring

## Abstract

Over the past decade, distributed acoustic sensing has been utilized for structural health monitoring in various applications, owing to its continuous measurement capability in both time and space and its ability to deliver extensive data on the conditions of large structures using just a single optical cable. This work aims to evaluate the performance of distributed acoustic sensing for monitoring a multilayer structure on a laboratory scale. The proposed structure comprises four layers: a medium-density fiberboard and three rigid polyurethane foam slabs. Three different damages were emulated in the structure: two in the first layer of rigid polyurethane foam and another in the medium-density fiberboard layer. The results include the detection of the mechanical wave, comparing the response with point sensors used for reference, and evaluating how the measured signal behaves in time and frequency in the face of different damages in the multilayer structure. The tests demonstrate that evaluating signals in both time and frequency domains presents different characteristics for each condition analyzed. The supervised support vector machine classifier was used to automate the classification of these damages, achieving an accuracy of 93%. The combination of distributed acoustic sensing with this learning algorithm creates the condition for developing a smart tool for monitoring multilayer structures.

## 1. Introduction

Structural health monitoring (SHM) is a set of techniques used to ensure the integrity of a structure with permanently installed transducers so that measurements can be taken constantly without interrupting the structure’s operation [1]. Furthermore, the structure’s condition is carried out by comparing current measurements with previous ones, a method known as baseline subtraction. Appropriate techniques for analyzing and identifying discontinuities, fissures, and cracks in materials are necessary for various applications, especially those capable of identifying these discontinuities early, preventing their integrity from being compromised. These techniques are particularly valuable when they can detect issues early, thereby preventing the compromise of structural integrity. By implementing continuous or regular monitoring techniques, the aim is to prevent unexpected structural failures or to identify early the appearance of damage to these structures [2,3]. The damage identification system in an SHM involves three main steps: signal monitoring, processing, and interpretation [4].

Monitoring a structure relies on sensors used to measure physical quantities. According to [5], these quantities can be classified into three categories: kinematic quantities (such as displacement, velocity, and acceleration), mechanical quantities (such as forces, deformations, and stress), and environmental quantities (such as temperature and wind). In recent decades, fiber optic sensors, especially fiber Bragg grating (FBG) [6], have gained ground in civil engineering applications for SHM [7]. Such popularity is mainly due to the intrinsic advantages of optical fiber, such as its dielectric constructive characteristics, lightness, dimensions, multiplexing capacity, wide bandwidth, and immunity to magnetic interference [8,9].

In recent decades, fiber optic sensors (FOSs) have gained ground in civil engineering applications for SHM [7]. Such popularity is mainly due to the intrinsic advantages of optical fiber, such as its dielectric constructive characteristics, lightness, dimensions, multiplexing capacity, wide bandwidth, and immunity to magnetic interference [8,9]. Point FOS can replace conventional sensors in the measurement of critical parameters monitored SHM, such as temperature, pressure, and strain [7]. The most commonly used FOSs in large structures are Fabry–Perot interferometers, fiber Bragg gratings (FBGs), and long-period fiber gratings (LPFGs) [10]. Another great advantage that optical sensors bring to the SHM is the possibility of being incorporated into the structure itself.

The class of fiber-optic sensors (FOSs) that has attracted significant attention for applications in SHM is distributed fiber-optic sensors (DFOSs) [11]. DFOS utilizes an optical fiber as a sensing element, enabling the measurement of strain and/or temperature along the entire fiber length, which can extend up to tens of kilometers [12]. This capability allows for the comprehensive monitoring of the entire length of the monitored structure [13]. Installing the same number of conventional point sensors would be unfeasible expensive, and laborious [14]. Additionally, the small diameter of the fiber-optic cable used for detection ensures the possibility of installing DFOS in limited spaces where conventional sensors cannot [15].

Among the various types of DFOSs, distributed acoustic sensing (DAS) has gained prominence in recent years for SHM applications [16]. DAS is based on Rayleigh backscatter and measures dynamic strain and temperature [13]. The first reported applications of DAS systems were in the oil and gas industry, for applications such as well integrity [17], and vertical seismic profile (VSP) acquisition [18]. Within the scope of SHM, applications of the DAS system have shown potential for rail track health monitoring [19], bridges [16], tunnels [20], roads [21], and dams [22].

This study aims to demonstrate a representative laboratory-scale test for monitoring multilayered structures. Following a successful proof of concept in the laboratory, this method could potentially be applied to large structures, such as dams and bridges, in the future. Two main results are presented: the detection of the mechanical wavefront propagated in the structure, measured by the DAS, and the use of a supervised classifier to automatically detect and classify different damages emulated in the multilayer structure based on the time and frequency analysis of the signals measured by the DAS system.

Ensuring the correct detection of the wavefront is essential for analyzing structures, as it greatly influences the recorded data and directly affects the determination of travel time in tomographic analyses [23]. Therefore, correctly characterizing the DAS system parameters (such as pulse width and pulse repetition rate) is crucial for guaranteeing accurate wavefront detection.

A significant challenge encountered when using the DAS system is the amount of data and the very high data acquisition speed required to extract the information of interest in the monitored structure. Depending on the length of the instrumented optical fiber, it is possible to reach an amount of data greater than 1 TB per day [24]. Processing and selecting important information from such a large amount of data, often in real-time, is challenging. Using efficient tools based on machine learning is an advantageous alternative for this purpose [25]. Furthermore, the amount of data generated in SHM is generally high, as analyses are performed with high frequency, sometimes even daily [1].

Using machine learning methods to extract features and classify damages in structures can be a promising tool to assist in processing the large amount of data generated by DAS for SHM. According to [26], feature extraction and pattern recognition techniques are the most crucial stages in the SHM process. In the same way, applying machine learning methods to analyze a multilayer structure and detect damage presents a viable alternative to techniques that require numerical modeling of the structure under analysis. Creating such models is often unfeasible and impractical due to the complexity of the structure, the number of layers, and its dimensions. Thus, this article presents a technique for extracting temporal and frequency characteristics of dynamic strain signals measured using DOFS, which is used as a supervised classifier for detecting damage in a multilayer structure.

This paper is structured as follows. Section 2 outlines the proposed methodology for reconstructing the mechanical wavefront generated within the structure, as measured by DAS, and damage detection in a multilayer structure using a supervised classification technique, support vector machine (SVM). Section 3 summarizes the obtained results. The main contributions of the study and future research directions are discussed in Section 4.

## 2. Materials and Methods

This section describes the materials and methods developed for two test conditions. Section 2.1 presents the experimental setup to reconstruct the mechanically propagated wavefront in rigid polyurethane foam (PUR) measured by DAS and fiber Bragg grating (FBG). Section 2.2 presents the experimental setup that emulates different damages in a multilayer structure. Based on the data measured from the four damage conditions simulated in this multilayer setup, supervised classifiers will automatically classify each type of damage. The instrumentation of both experimental setups, data acquisition, and processing are described in the following section.

### 2.1. Experimental Setup for Mechanical Wavefront Analyses

**Figure 1:**
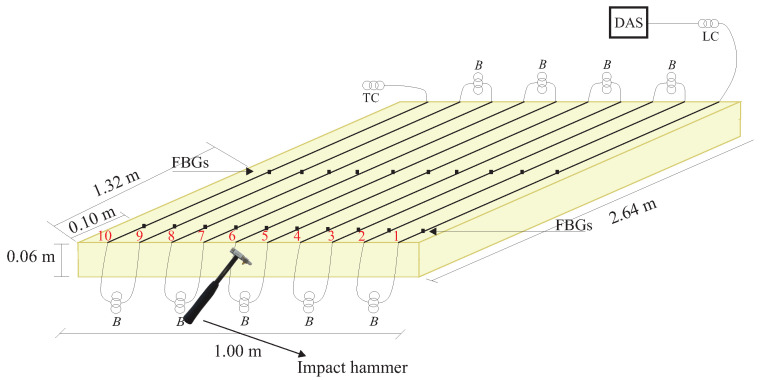
The arrangement of optical fibers and FBGs in a PUR foam slab used to reconstruct the excited wavefront at the midpoint of the slab width by a modal hammer.

This test was designed according to Figure 1 to excite a mechanical wave propagating through a PUR and measuring it using optical fiber sensors. To reconstruct the generated wavefront, it is necessary to reduce the influence of environmental noise. The PUR slab was suspended using nylon strings attached to the laboratory ceiling. The slab was suspended by nylon threads and tied by five points to distribute the weight of the slab evenly. The PUR foam has the following dimensions: 2.64 m × 1.00 m × 0.06 m and a density of 40 kg/m^3^. The choice of PUR material is due to its heterogeneous characteristics and low density, making it possible to more accurately replicate a situation similar to the propagation of low-speed seismic waves.

The tests were initially carried out on just one layer to facilitate the understanding of the DAS system’s measurement method for reconstructing mechanical waves. An impact hammer generated a mechanical wave in the thickness of the PUR foam slab. The impact occurred at the midpoint of the slab’s width, as depicted in Figure 1.

For wavefront detection, ten single-mode optical fibers (SM), with a silica core, numerical aperture NA=0.14, and attenuation equivalent to 0.18 dB/km were instrumented along the length of the PUR foam, as shown in Figure 1. The first fiber was instrumented at a distance of 0.05 m from the slab’s width. Each fiber was spatially instrumented separately at intervals of 0.10 m from one another. To distinguish the signals measured in each fiber with the DAS, separation buffers consisting of 20 m of optical fiber were utilized. The fibers used in the tests are single-mode acrylic-coated optical fibers and were affixed to a structure using cyanoacrylate-based glue.

FBGs were used as reference sensors to determine the wavefront of the mechanical wave propagating in a setup generated by an impact hammer. Two optical fiber patch cords, each containing 10 FBGs, were installed perpendicular to the length of the PUR foam slab. Each FBG was installed alongside the optical fibers of the DAS system. The FBGs were inscribed in SM GF1 optical fibers, with a silica core, and numerical aperture NA=0.13 using the phase mask technique and an excimer laser (Xantos XS 500-193 nm-XS-L from the company Coherent, Inc, manufactured in Saxonburg, PA, USA) with pulsed emission up to 500 Hz at the Multi-user Photonics Laboratory of the Federal University of Technology—Paraná. The Bragg wavelengths used are spaced between 1526 nm to 1560 nm, with a reflectivity of 75% and full width at half maximum of approximately 0.3 nm. The first patch cord was installed 0.10 m from the beginning of the slab’s length, while the second patch cord was installed 1.32 m from the slab. Each FBG is spaced every 0.10 m along the patch cords.

In Figure 1, the optical fibers are represented with black lines in the PUR foam slab, each indicated by its respective number, and the black squares represent the FBGs. The buffers for separation, denoted by the letter B in the diagram, are utilized between each sensor fiber. An optic fiber launch coil (LC) connects the experimental array to the DAS. At the array’s end, a termination coil (TC) serves as a buffer to differentiate the last sensing fiber from the end of the optical array.

The DAS interrogator unit is based on the traditional ϕ-OTDR heterodyne detection topology, described in [27]. The repetition rate of the DAS system used is 100 kHz, the pulse width is 30 ns, and the spatial resolution defined by the gauge length equals 3 m. The FBG signals were acquired using the SM130 optical interrogator, manufactured by Micron Optics, with a sample rate of 1 kHz.

The high pulse repetition rate guarantees the detection of the wavefront generated during excitation. In the ϕ-OTDR system, the measured phase must be unwrapped using unwrapping methods. However, these methods are constrained by the Itoh criterion [28], which demonstrates that the phase variation cannot exceed π radians. Otherwise, distortions in the measured signal will occur. The Itoh criterion limits the amplitude of the measured strain and defines the minimum pulse repetition rate that should be used. In [29], it is explained that the measured amplitude without distortion is increased with an increase in the pulse repetition rate.

**Figure 2:**
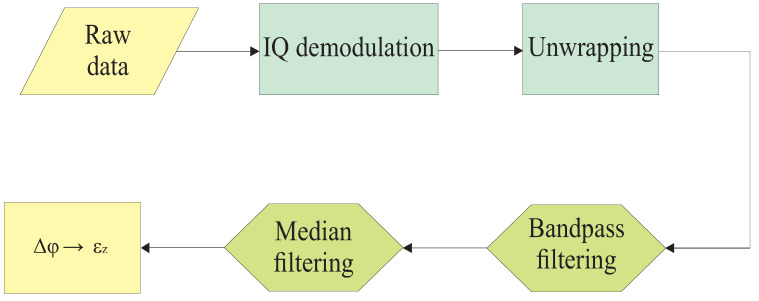
Flowchart of the processing to classify discontinuities in the multilayer structure.

For the reconstruction of the wavefront, it is necessary to obtain the strain measured by each optical fiber instrumented in the multilayer structure. The flowchart presented in Figure 2 details the procedures, from pre-processing the raw data measured by the DAS to converting the measured phase into strain. The phase information of the backscattered signal is extracted from the in-phase and quadrature demodulation (IQ) of the signal, followed by phase unwrapping. After phase unwrapping, the phase difference Δϕ between two neighboring regions is obtained, separated by gauge length $L_{g}$.

The Δϕ matrix was filtered from 3 to 1.5 kHz using a second-order bandpass Butterworth filter to select only the frequencies of interest from the signals. The median filtering was applied in spatial and temporal domains to suppress undesirable noise [30].

Subsequent to filtering, the demodulated phase is converted into strain. The phase difference is related to the longitudinal strain $\varepsilon_{z}$ experienced at a specific location along the length of the optical fiber at a given moment in time by the Equation (1) [31]:

(1) $$\Delta \phi =\frac{\left(4\pi n_{eff}L_{g}\varepsilon_{z}\xi \right)}{\lambda },$$

where $n_{eff}$ is the refractive index of the fiber equivalent to 1.4682 at 1550 nm, ξ is the photoelastic constant usually taken as 0.78 at 1550 nm [31], and λ is the center wavelength of the probe pulse.

### 2.2. Multilayer Experimental Setup

**Figure 3:**
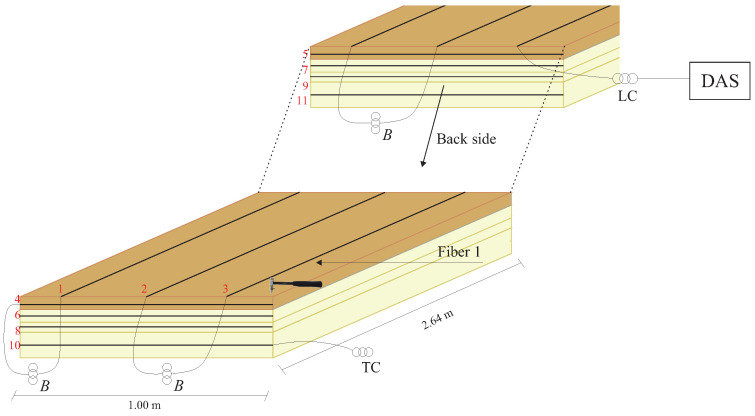
The experimental setup composed of four layers (one MDF and three PUR foam) instrumented with eleven optical fibers for detecting acoustic waves to characterize the response of DAS to different damage conditions.

The experimental setup to emulate different damages consists of four layers composed of two distinct materials: medium-density fiberboard (MDF) and rigid polyurethane foam (PUR) (Figure 3). The first layer is MDF with dimensions of 2.64 m × 1.00 m × 0.025 m. The second and third layers are made of polyurethane foam with dimensions of 2.64 m × 1.00 m × 0.03 m, and the fourth layer is made of polyurethane foam with dimensions of 2.64 m × 1.00 m × 0.06 m. The choice of these two materials is due not only to their dimensions but also to their difference in density. Polyurethane foam has a low density, around 40 kg/m^3^, while the density of MDF is approximately 750 kg/m^3^. This contrast in density is interesting for evaluating the behavior of acoustic waves propagating in a multilayer structure, especially when the structure is compromised with some damage.

The mechanical waves propagated through the structure were generated with an impact hammer, excited on the MDF board at the beginning of the slab’s length, as illustrated in Figure 3, represented by a red arrow. These mechanical waves were measured with DAS, using eleven SM optical fibers instrumented in the structure. Three of them (Fibers 1, 2, and 3) were instrumented on the MDF board along the length of the board and equally spaced at 0.2 m intervals. Another four optical fibers (Fibers 4, 6, 8, and 10) were instrumented on the side of each layer along the width of the structure. On the other side of the slabs, along the width of each one, four optical fibers (Fibers 5, 7, 9, and 11) were also instrumented. Fibers 4 to 11 were instrumented to measure the propagation speed of the mechanical wave in the structure.

The optical fibers are represented by black lines in the structure as shown in Figure 3, each indicated by its respective number. The fibers used in the tests were single-mode acrylic-coated optical fibers and were affixed to a structure using cyanoacrylate-based glue.

To distinguish the signal measured by each optical fiber, separation buffers of 20 m were used between the fibers. They were accommodated between acoustic insulation foams to prevent the buffers from measuring the excitation signals. The DAS system was set with a pulse repetition rate of 100 kHz, a pulse width of 30 ns, and a gauge length of 3 m.

**Figure 4:**
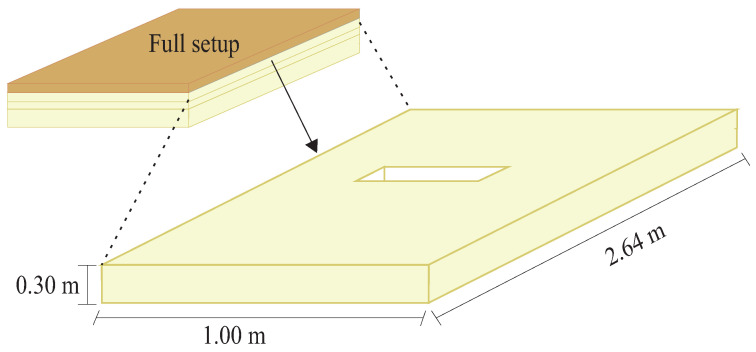
Location and dimensions of the emulated damages in the PUR foam, which is the second layer of the structure under analysis.

To evaluate the response of the DAS system to different damages in a multilayer structure, it was proposed to analyze two damages with different densities in the second layer of the structure, namely, in the first PUR foam. The first considered damage consists of a rectangular cutout measuring 0.10 m × 0.26 m × 0.03 m at the center of the PUR foam, as depicted in Figure 4. This cutout in the foam slab emulates a void filled with air. The second damage emulated was to fill this cutout with water. Thus, discontinuity with two materials of different densities is characterized by discontinuity with air, equivalent to 1.225 kg/m^3^, and discontinuity with water, with a density of 997 kg/m^3^.

**Figure 5:**
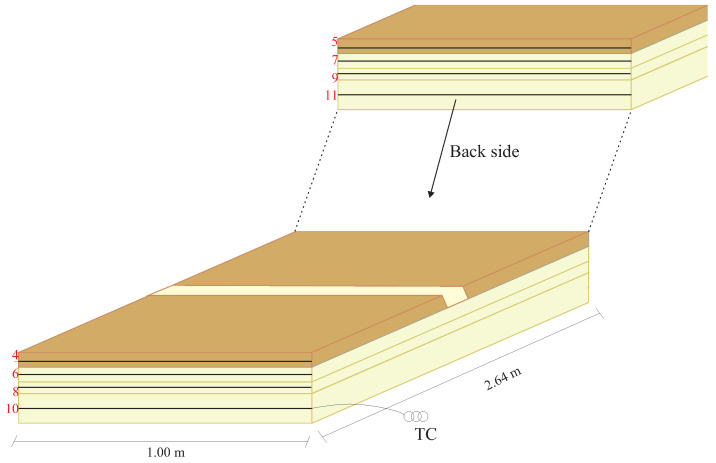
Representation of the emulation of a crack on the MDF board, which is the third damage emulated.

A third damage condition was also considered: a crack running along the entire width of the structure’s MDF board, with a thickness of approximately 0.003 m, as shown in Figure 5. This damage emulates a crack in the first layer of the structure throughout the board’s thickness, so the MDF was divided into two parts to simulate severe damage.

No fibers were used on the MDF board in this damage scenario. Thus, only eight fibers were used to analyze this condition. It is essential to highlight that the experiments conducted for this condition were carried out with the other three layers of the structure (PUR foam slabs) undamaged, without the damages mentioned in Figure 4.

As the main objective of these tests with different damages in the multilayer structure is to use a supervised classifier to detect these damages automatically, it is necessary to create a dataset. Five repeated tests were conducted for each structure condition considered in this study to characterize the DAS system response and identify the difference in detected signals among the four conditions under analysis. The tests induce excitation with an impact hammer on the MDF board near Fiber 1, as depicted in Figure 3.

**Figure 6:**
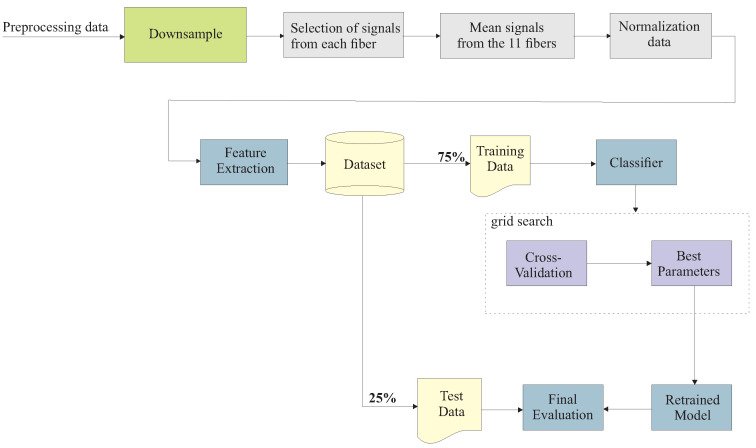
Flow diagram of the algorithm to classify different damages in the multilayer structure.

For the analysis of measured data in the multilayer structure, the same preprocessing presented in Figure 2 was used in all tests carried out in the multilayer structure. The steps used in data processing to classify damages in the structure are shown in Figure 6.

The signals were downsampled to the repetition rate equal to 10 kHz. This downsample was used because the frequency range of interest is small; frequencies up to 500 Hz were measured in the structure. The need to use a high pulse repetition rate was only to avoid errors in unwrapping. Therefore, the downsample at this processing stage would not result in losses in data analysis. Subsequent to the downsampling, the region of each optical fiber instrumented in the structure was determined.

The signals detected by the DAS were acquired by a 500 MS/s acquisition board, which implies that the system’s sampling resolution is 0.2 m. This means that for every 1 m of the gauge length, there are five traces sampled by the DAQ. As the gauge length used in the experiments is 3 m and the dimensions of the analyzed setup allow for only one sensor point, we have 15 traces for each of the instrumented fibers. The mean of the signals was calculated to improve the signal-to-noise ratio (SNR) because there is only one measurement point on the structure.

Then, the feature extraction was made using the Time Series Feature Extraction Library (TSFEL) library [32]. The created dataset consists of 205 samples with 389 features each. The TSFEL library was chosen for feature extraction as it can extract information from the data time, frequency, and statistical domains. As the crack condition on the MDF board has only eight optical fibers, the dataset created is unbalanced; this class presents fewer samples than the other 3 (undamaged, air discontinuity, and water discontinuity). Given this, the metric chosen to evaluate the accuracy of the dataset is an F1-macro score.

For the classification of damages in the multilayer structure, the machine learning model used was SVM. The SVM is a machine learning algorithm based on the Statistical Learning Theory initially proposed for classification based on the concept of an optimal separation hyperplane to maximize the separation margin between classes, as Vapnik proposed in 1995 [33]. For non-linear problems, the classifier’s objective is to find a hyperplane of maximum separation between classes, now using a kernel to transform the feature space.

To determine the best parameters of the classifier, grid-search, and stratified k-fold cross-validation techniques were employed with an independent test set [34,35]. The dataset was separated into 70% for the training set and 30% for the test set. Following the selection of the best parameters, the classifiers were re-trained. Finally, the test set was used to evaluate their performance.

## 3. Results and Discussion

### 3.1. Detection of the Mechanical Wavefront

**Figure 7:**
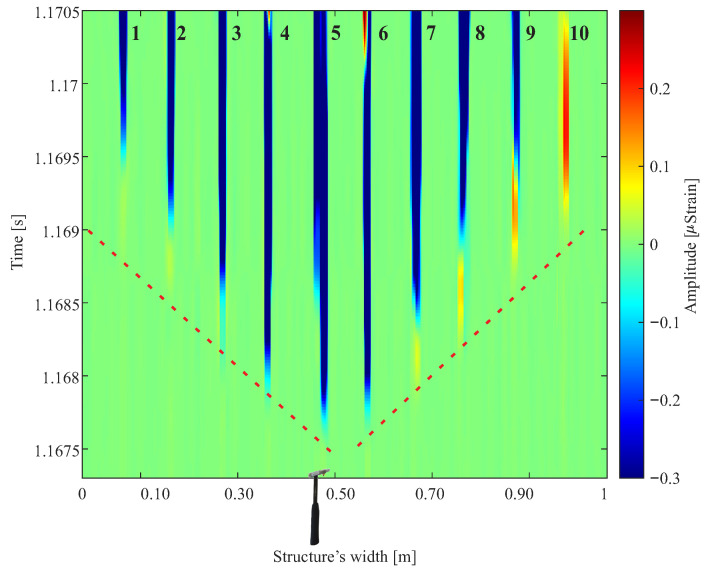
Wavefront detected by DAS in the setup of Figure 1. A modal hammer generated a mechanical impact in the middle of the width of the PUR foam.

The wavefront detected by the DAS is shown in Figure 7. A color map was utilized to illustrate the wavefront, with the *x*-axis denoting the length of the PUR foam, the *y*-axis representing the propagation time of the mechanical wave in a PUR foam, and the strain intensity in each fiber being depicted by the color scale on the *z*-axis.

The arrangement of the fibers makes it possible to understand and reconstruct the wavefront generated from the excitation of the modal hammer in the middle of the width of the PUR foam. The detected wavefront has a spherical behavior because the wave is generated close to the sensors.

Preliminary tests using lower pulse repetition rates, such as 10 kHz, 30 kHz, and 50 kHz, demonstrated that unwrapping errors occurred due to the high strain amplitude caused in the PUR slab and, therefore, errors in wavefront detection occurred. With 50 kHz it was possible to correctly reconstruct the wavefront for a layer depending on the deformation amplitude applied to the slab. However, for the multilayer structure, the ideal repetition rate was equal to 100 kHz.

Fibers 5 and 6, located in the center of the PUR foam, are sensitized first since they are closest to the excitation generated by the impact hammer. Then, the other fibers detect the wave until fibers 1 and 10, which are at the ends of the PUR foam, detect the wave. As excitation was conducted at the midpoint of the width along the length of the PUR foam, symmetry is observed in the detected signal between the instrumented fibers spanning the two halves of the foam.

**Figure 8:**
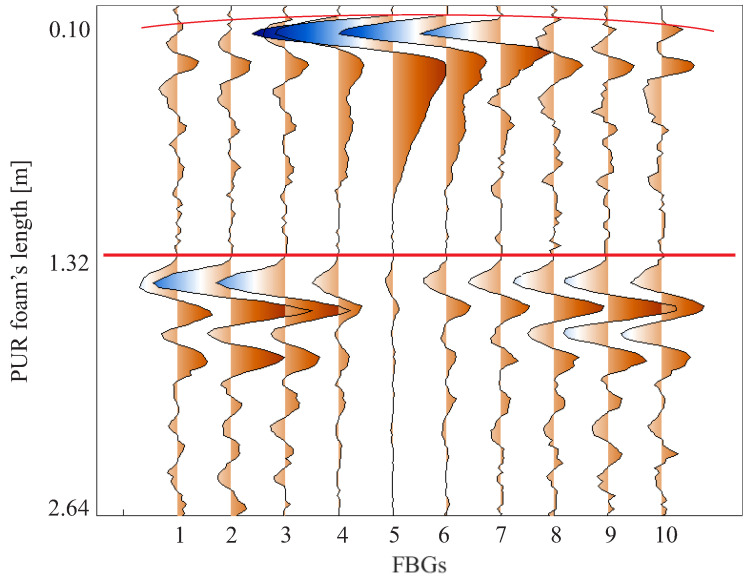
Wave propagation detection in the setup of Figure 1 using FBGs. The spherical wavefront is demonstrated at the plot’s beginning (0.10 m), and the central FBGs (1.32 m) measure the plane wave arriving in the middle of the PUR foam.

FBGs, which are point strain sensors, were used as a reference. The first 10 FBGs, instrumented 0.10 m from the start of the foam length, detect the same spherical wavefront behavior as detected by the DAS. The FBGs instrumented in the middle of the foam length demonstrate that the wave behavior after 1.32 m is already plane since the FBGs simultaneously detect the propagated wave. This behavior is shown in Figure 8. The DAS cannot detect the wave becoming flat at half the length of the PUR foam slab due to its spatial resolution, which allows only one measurement point due to the dimensions of the foam.

The central FBG (FBG 5 in Figure 8) presents the largest measured deformation amplitude since the excitation was very close to it. FBGs 4 and 6 show a measured strain that is smaller but larger than the strain measured by the remaining FBGs. So, in addition to the measurement instant of each FBG being different due to the sphericity of the mechanical wavefront, the amplitude of the detected wave is also different since the closer FBGs are more sensitized by the wave than the FBGs further away from the location of wave excitation.

### 3.2. Discontinuities Analysis in Multilayer Structures

**Figure 9:**
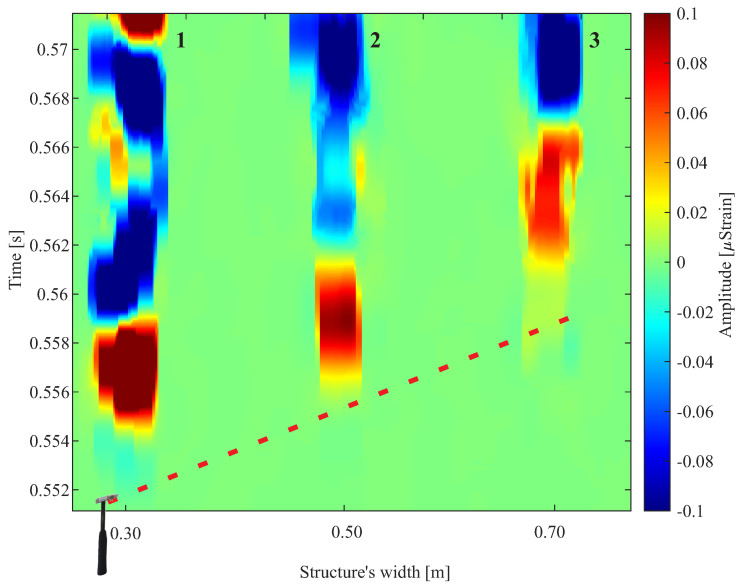
The spherical wavefront was detected by DAS using three instrumented optical fibers on the MDF board in the setup of Figure 3. The presented wavefront corresponds to an MDF board excitation generated with an impact hammer near Fiber 1.

Following the excitation initiated with an impact hammer positioned as shown in Figure 3, Figure 9 illustrates the three optical fibers instrumented on the MDF reconstructing this spherical wavefront. Another characteristic observed is that the intensity of the measured signal is stronger in Fiber 1. As it propagates towards the end of the slab width, the intensity decreases, as observed in Fibers 2 and 3. This behavior also indicates that the wave measurement is spherical.

Both Figure 7 and Figure 9 demonstrate the ability of the DAS system to differentiate signals from instrumented fibers that are spatially separated by just a few centimeters due to the use of separation buffers utilized and isolated in the measurement environment of interest. This result demonstrates the feasibility of employing fibers or optical cables in strategic configurations to monitor critical areas in structures, enabling finer resolution of the targeted locations.

Another characteristic extracted from the time domain data is the propagation speed of the mechanical wave generated in the experimental setup. The speed was calculated considering the MDF board’s length (2.64 m) and the wave propagation time between Fibers 4 and 5, instrumented in the thickness of the two sides of the MDF board, as indicated in Figure 3. The position of these fibers was made to allow measurement of the wave propagation speed in the structure. Fiber 4 is used as an optical trigger, measuring the moment at which excitation is generated on the MDF board using the impact hammer. Fiber 5, installed on the other side of the MDF, is used to measure how long the wave took to propagate to the end of the experimental setup.

For the structure without damage, the average speed for the five tests carried out is equal to 1590 m/s; for the structure with air discontinuity, the resulting speed is 996 m/s; for the structure with water discontinuity, the speed found is equal to 1250 m/s while for the structure with the crack in the MDF, the average speed obtained is equal to 250 m/s. The speed result for each of the conditions analyzed is related to the net density of the structure since air and water have different densities due to the severity of the damage.

The reduction in wave propagation speed in the structure with the crack in the MDF is because the emulated damage divided the MDF board into two parts, and, therefore, the only wave propagation path was through the foam. As the generated damage has a critical severity, so do the structure’s mechanical properties, such as stiffness and density.

**Figure 10:**
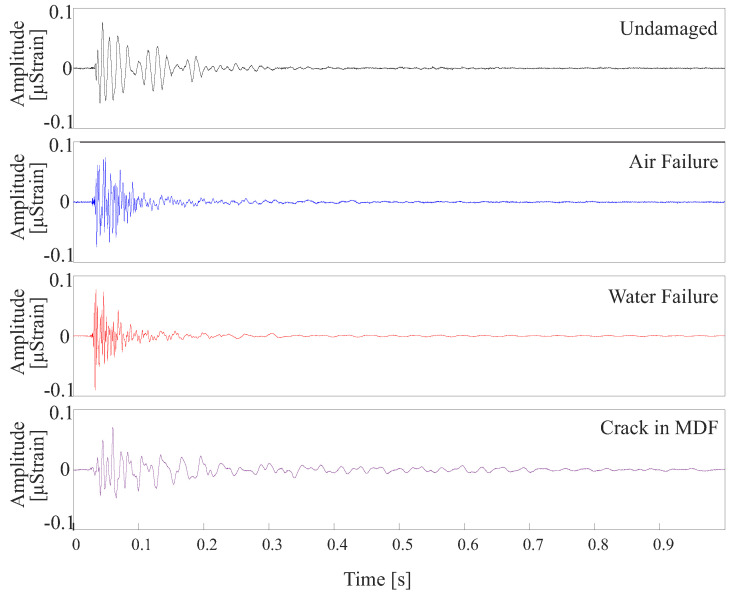
Comparison of the signal over time measured by fiber 5 for the four conditions emulated in the multilayer structure.

By inserting different damages in the experimental setup, it is possible to observe differences in the signals measured by the same fiber. In Figure 10, the Fiber 5 signal is presented for the four conditions analyzed in the structure. The choice of this fiber to demonstrate the detected signals is due to the fact that it is the only fiber instrumented in the superficial layer of the structure for the four conditions of the analyzed structure.

The envelope of the measured wave is visibly different. Air and Water discontinuity conditions present an envelope with a higher frequency than the undamaged and cracked in MDF conditions. Furthermore, the propagation duration of the mechanical wave is also different, probably due to the wave reflections resulting from the encounter of the direct wave with the damaged points in the setup.

In addition to temporal analysis, frequency analysis can demonstrate differences between the conditions analyzed in the structure. The occurrence of damage in the structure can change its natural frequencies [36]. Furthermore, these changes are related to the nature of the damage, location, and severity. In this way, the power spectral density (PSD) analysis can extract meaningful information about the structure’s condition, as it allows an understanding of which frequencies contribute most to the total power of the signal for each of the conditions analyzed and highlights the differences between them [37].

The PSD analysis of an undamaged structure will present a specific response with frequencies related to the structure’s vibration modes. When damage is inserted into this structure, the mechanical and acoustic parameters are changed, and consequently, the PSD of the signal will be different.

**Figure 11:**
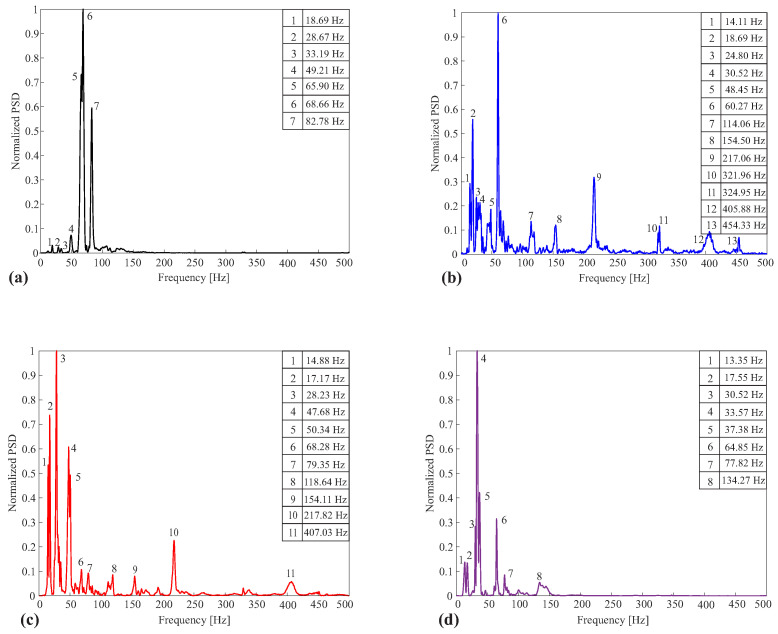
Comparison of the PSDs of the four conditions emulated in the experimental setup. (**a**) PSD of Fiber 5 in the undamaged setup. The spectrum shows only components at frequencies lower than 100 Hz in the undamaged structure. (**b**) PSD of Fiber 5 in the setup with air discontinuity. In this condition, higher frequency components arise due to water discontinuity in the structure. (**c**) PSD of Fiber 5 in the setup with water discontinuity. In this damage condition, the higher amplitude frequencies disappear. (**d**) PSD of fiber 5 in the setup with a crack in the MDF board. It is possible to notice that the frequency range with energy information is similar to that of the undamaged structure.

Damage can change the resonant frequencies of the structure and even introduce new vibration frequencies that did not exist in the structure without damage. To exemplify the comparison of the response measured by the DAS system for the four conditions of the structure under analysis, Figure 11a–d present the PSD of Fiber 5 instrumented in the MDF board of the structure. The PSD presented results from the average of the five PSDs obtained in the five tests carried out for each condition.

It is important to point out that for each fiber, the PSD will have a different response since the position of the instrumentation of each fiber favors the measurement of one direction of the mechanical wave. Figure 11 presents the response of the undamaged structure of the signals measured by Fiber 5. There are few components with relevant amplitude in the analyzed spectral range, with 68.66 Hz being the most significant.

The spectra presented in Figure 11b,c correspond to air and water discontinuities, respectively. These are the two spectra exhibiting the highest quantity of frequency components, likely due to the discontinuities’ positions in the second layer of the structure, providing more paths for wave reflections to propagate within the structure.

The most significant frequency components are 60.27 Hz and 28.23 Hz for air and water discontinuities, respectively. In the PSD of the structure without any damage, the highest frequency component is 82.78Hz. However, in the PSDs of the structures with air and water discontinuities, the highest frequency components are 454.33 Hz and 407.03 Hz, respectively.

The PSD of the crack in MDF presented in Figure 5 has the most significant component at 33.57 Hz, and its highest frequency component is 134.275 Hz. Compared to the spectra of air and water discontinuities, the more well-behaved pattern is noticeable, with fewer peaks in the spectrum. A possible explanation is that the crack in MDF occurs along the entire width of the board, resulting in its division into two. As a result, the energy of the propagating wave, the only path for transmitting this wave energy to the second half of the MDF is through the PUR foam slabs, which have a smaller density and a higher capacity to absorb energy than MDF.

Differences in the signals for each analyzed condition are noticed when analyzing the signal in the time and frequency domain. However, it is costly to perform this manual analysis. Therefore, supervised classifiers were used to automatically classify each condition emulated in the experimental setup.

The training of the supervised SVM model was carried out with 70% of the dataset formed with the 389 features extracted by the TSFEL library. From grid search and cross-validation, the parameters selected for the SVM are regularization parameter *C* equal to 46, radial basis function (RBF) kernel, and the γ parameter equal to 0.0026.

For the test dataset (30% of the original dataset), the F1-macro score obtained equals 85%. The test set confusion matrix is presented in Table 1. The score achieved is satisfactory since the dataset used for training has few samples for each analysis condition and a wide variety of signals since signals from all instrumented optical fibers were used. As explained, each instrumented fiber presents a different response since the characteristics of the propagated wave change in each layer and position of the fiber due to the DAS response.

One point to highlight is that this instrumentation in all layers of the structure is not possible in existing structures; it would only be possible if the fibers were instrumented during their construction. Therefore, thinking about a more realistic application that can be used in existing structures, a new approach to training the SVM classifier was proposed. This new approach uses only the fibers instrumented on the MDF board; given that when DAS is used for SHM in actual structures, it is advantageous to simplify instrumentation by installing the optical cable on this part of the structure.

The three fibers (Fibers 1, 2, and 3) instrumented on the structure were used for the new dataset. However, as the crack in the MDF condition did not consider these fibers, only the undamaged, air discontinuity, and water discontinuity conditions were analyzed. As there are 3 fibers on the MDF board and 5 test repetitions, the total dataset, with the three classes, consists of 45 samples (15 for each condition) and 389 features.

Again, grid search and cross-validation were performed to define the parameters. The new SVM parameters are *C* equal to 50, RBF kernel, and γ equal to 0.0026. In this case, the training dataset is 75% of the full dataset, and the testing set is 25%. For the test dataset, the accuracy achieved was 93%.

For this analysis, accuracy was used instead of the F1-macro score because, in this case, the dataset is balanced, which is the appropriate metric. Table 2 presents the confusion matrix for the set of tests for the three conditions analyzed (undamaged, air discontinuity, and water discontinuity).

In this way, using machine learning techniques for feature extraction and fault classification in structures is an interesting approach. This is primarily due to the reduced processing time in more sophisticated algorithms for fault classification and visual data analysis.

In this case, the accuracy was higher (93%) because even with few samples making up the dataset, the variability of the signals that make up the dataset is lower. Furthermore, the three optical fibers used to measure the tests for this dataset are instrumented in the same region (on the MDF board). Therefore, the characteristics of the signals are similar. This result demonstrates that the proposed tool for classifying structural conditions in conjunction with the DAS system for SHM has potential, mainly due to the high accuracy rate in classifying conditions using optical fibers instrumented only on the structure.

Furthermore, another interesting result is that the faults detected are in internal layers of the structure, demonstrating the validation of the tool for detecting faults at an early stage. This way, using machine learning techniques for feature extraction and fault classification in structures is an interesting approach. This is primarily due to the reduced processing time in more sophisticated algorithms for fault classification and visual data analysis.

The results obtained from tests carried out on the laboratory-scale multilayer structure demonstrate that combining DAS with the supervised classifier SVM, trained with temporal and frequency characteristics extracted from the structure, is a promising solution for monitoring large structures and for SHM. Validating a new technique through laboratory tests is a fundamental step to demonstrate the performance of a proposed technique before instrumenting a large structure. In [38], the authors demonstrate the effectiveness of using DFOS through small-scale laboratory experiments, using a smart composite deck panel to be implemented in the future in the FRP bridge. The FRP sandwich deck panel consists of multilayer lower and upper FRP laminates and a PUR core placed between faces. The longitudinal and transverse optical fiber instrumentation was arranged to enable distributed strain and temperature measurement. Work like this demonstrates the importance of conducting laboratory experiments to calibrate and validate the sensor used and the processing methods.

## 4. Conclusions

This work used the DAS system to detect and reconstruct the mechanical wavefront generated in a PUR foam. The optical fiber instrumentation of the DAS system was also demonstrated in a multilayer structure of laboratory dimensions to measure and characterize the signals measured in this structure with different types of damage.

Regarding the detection of the wavefront, the results demonstrate that due to the instrumental arrangement of the optical fibers, the DAS can reconstruct the spherical wavefront propagated by the PUR foam. The FBGs used as a reference confirmed that the mechanical wavefront generated by an impact hammer on the PUR foam was initially spherical, and that it became flat halfway along the length of the foam.

Relating to the analysis of different damages in the multilayer structure, it was demonstrated that it is possible to identify differences in the signals measured for each of the four conditions analyzed in the time and frequency domains. However, to facilitate the automatic processing and classification of this data, it was demonstrated that machine learning techniques could be an advantageous and attractive alternative for multilayer structure damage analysis since the accuracy achieved was equivalent to 85%.

Considering applications for actual structures, a second damage classification approach using SVM was presented. Using only the optical fibers instrumented on the MDF board, the SVM classifier classified three conditions of the analyzed structure (undamaged, air discontinuity, and water discontinuity), reaching an accuracy of 93%. This result demonstrates that with simplified instrumentation of optical cables only on the surface of the actual structure, it is possible to use machine learning tools to detect faults at an early stage and in the internal layers of the structure.

In this way, using machine learning in conjunction with feature extraction reduced the complexity of data analysis, making it a useful tool to use in conjunction with DAS for SHM. Carrying out tests in the laboratory before instrumenting large structures is essential as it allows for a greater understanding of both the sensor used and the data acquired. Due to these tests, it was possible to understand that with a specific instrumental arrangement with optical fibers, the spatial resolution of the DAS system in centimeters can be improved. It was demonstrated that using separation buffers between the measurement fibers allows the DAS to distinguish signals measured by fibers separated by only 0.10 m since these buffers were accommodated in locations isolated from the excitations carried out for the tests. The proposed supervised classifier enables the evaluation of the technique’s application to actual structures, such as dams. Techniques that automatically classify dam discontinuities can improve dam safety and reliability, thereby contributing to risk mitigation and protecting affected communities.

The main advantages of the proposed technique include the simplicity of instrumentation with a single optical cable capable of monitoring tens of kilometers and the application of a machine learning technique that identifies damages in the internal layers of the analyzed structure, using a sensor installed on the structure’s surface. Furthermore, using SVM facilitates data analysis, as damage detection is automated, and the pre-processed data extract only the relevant features for classifier training.

## Figures and Tables

**Table 1:** Confusion matrix of the unbalanced test set of four classes of the SVM classifier using the TSFEL for feature extraction.

		Predicted Class			
True class	Undamaged	11	0	0	0
	Air Discontinuity	0	8	3	0
	Water Discontinuity	0	0	9	2
	Crack in MDF	0	0	1	7
		Undamaged	Air	Water	Crack

**Table 2:** Confusion matrix of the unbalanced test set of three classes of the SVM classifier using the TSFEL for feature extraction.

		Predicted Class		
True class	Undamaged	4	0	0
	Air Discontinuity	0	5	0
	Water Discontinuity	1	0	4
		Undamaged	Air	Water

## Data Availability

Dataset available on request from the authors.
